# PERCEPTIONS, ACCEPTABILITY, EXPECTATIONS, AND CONCERNS OF SMART HOME TECHNOLOGIES AMONG OLDER ADULTS

**DOI:** 10.1093/geroni/igz038.3229

**Published:** 2019-11-08

**Authors:** Amy Guan, Hannah C Dannewitz, Lauren Stratton, Jennifer Margrett, Walter R Boot, Neil H Charness, Balaji Narasimhan

**Affiliations:** 1 Iowa State University, Ames, Iowa, United States; 2 Florida State University, Tallahassee, Florida, United States

## Abstract

Optimal aging in place has become a common preference among older adults to maintain identity and independence, thus smart home technologies are increasingly utilized to achieve these goals. However, disconnect may exist between potential technological benefit and perceptions of acceptability and usability (Lee & Coughlin, 2015). We assessed perceptions of adults aged 50+ (range 50-90 years) to analyze their priorities and ultimate acceptability of smart home technology. Data were collected through surveys, focus groups, and case study interviews. Three major themes emerged regarding smart home utilization: benefits, concerns, and expectations. Participants endorsed smart home technologies (e.g., sensors, telehealth devices) and identified benefits, such as the promotion of optimal aging (e.g., maintaining independence, staying active, safety). However, responses also reflected concerns about privacy, ease of use, and amount of control. Expectations regarding smart homes included more mobility, efficiency, and safety within the home. One participant described technology as having “options [that] are exhausting, but also exciting.” Survey responses (n=30) were analyzed to understand participants’ familiarity with smart home technologies, including: nanotechnology (10.7%), smart showers (42.9%), home sensors (70.4%), telehealth (74.1%), smart appliances (71.4%), personal sensors (81.5%), and voice-activated devices (96.4%). Additionally, respondents indicated their willingness to implement these technologies to maintain and/or improve their daily functioning: nanotechnology (53.8%), smart showers (28.6%), home sensors (66.6%), telehealth (81.5%), smart appliances (40.0%), personal sensors (55.5%), voice-activated devices (64.3%). Discussion focuses on the priorities and needs older adults express regarding technology utilization and the implications for person-centered design and implementation of future smart home technologies.

